# Supplementary figures and images for: The single-species metagenome: subtyping Staphylococcus aureus core genome sequences from shotgun metagenomic data
Source: PeerJ. 2016 Oct 18;4:e2571. doi: 10.7717/peerj.2571 (PMC5075713; doi:10.7717/peerj.2571)

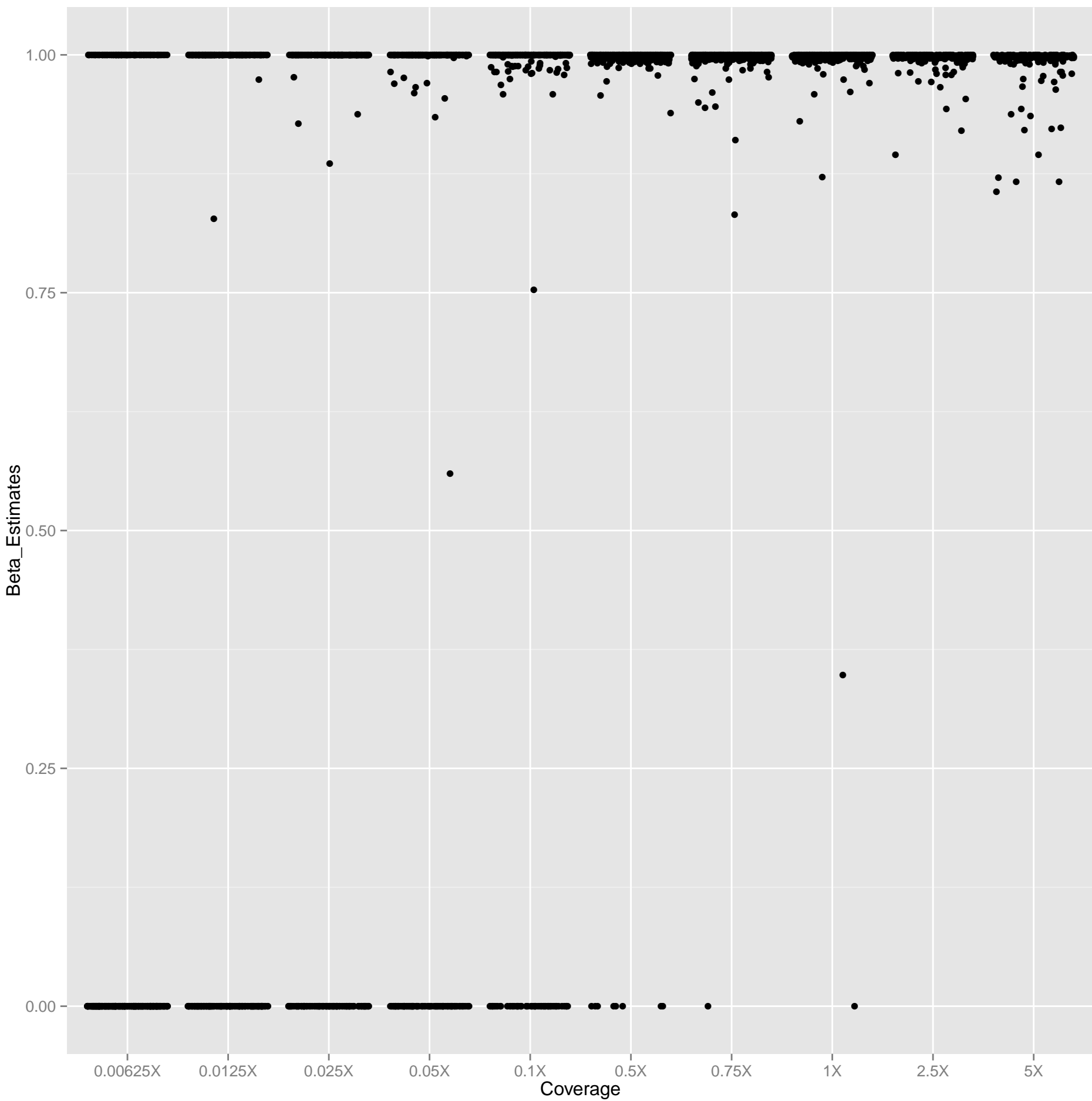

Supplement: Figure S1 [file peerj-04-2571-s001.pdf]

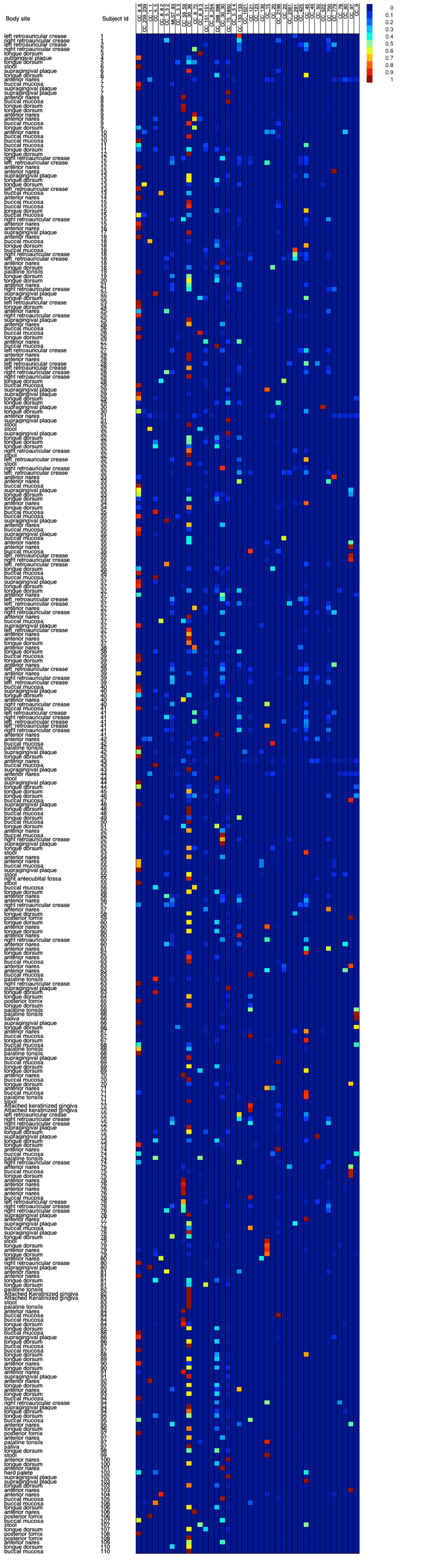

Supplement: Figure S2 [file peerj-04-2571-s002.pdf]

Cov. &gt; 0.025 by body site

S. aureus coverage

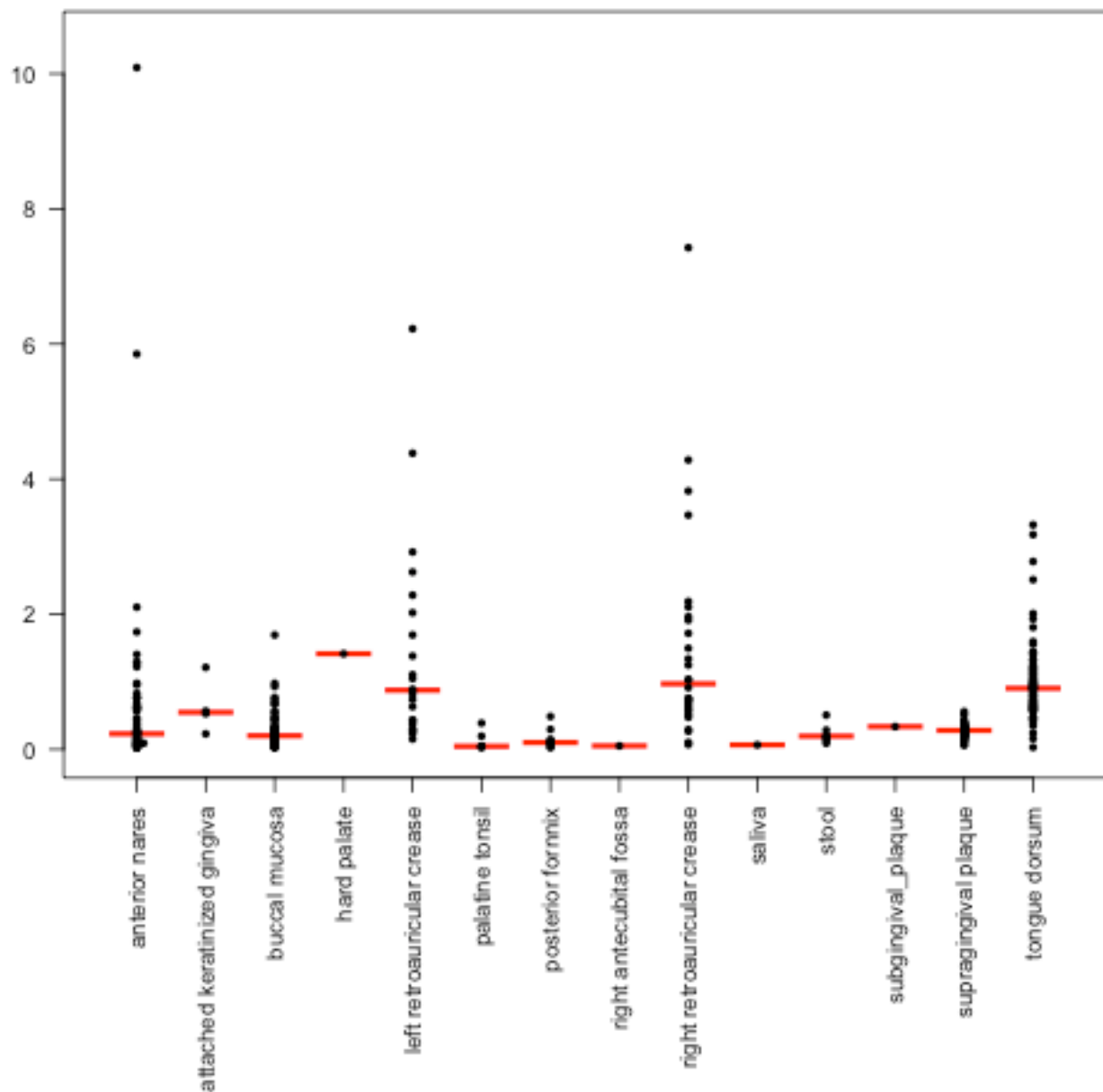

Supplement: Figure S3 [file peerj-04-2571-s003.pdf]
